# Near-Complete Genome Sequence of a Fish Nervous Necrosis Virus Isolated from Hybrid Grouper in China

**DOI:** 10.1128/MRA.01453-19

**Published:** 2020-04-09

**Authors:** Peng Jia, Xiaoqi Chen, Jiajie Fu, Meisheng Yi, Wenbo Chen, Kuntong Jia

**Affiliations:** a School of Marine Sciences, Sun Yat-sen University, Guangzhou, Guangdong, China; b Southern Marine Science and Engineering Guangdong Laboratory (Zhuhai), Zhuhai, Guangdong, China; c Dalian Modern Agricultural Production Development Service Center, Dalian, China; KU Leuven

## Abstract

The genome sequence of nervous necrosis virus strain HGN1910, isolated from hybrid grouper (Epinephelus fuscoguttatus♀ × E. lanceolatus♂), was cloned, sequenced, and characterized. Two near-complete gene segments were obtained, RNA1 and RNA2. Phylogenetic analysis shows that the virus belongs to the red-spotted grouper nervous necrosis virus genotype of betanodavirus.

## ANNOUNCEMENT

Nervous necrosis virus (NNV), also called betanodavirus, is a worldwide epidemic pathogen of fish (1). It can affect more than 120 kinds of different cultured and wild fishes in the larval and adult stages (2). Once infected with NNV, fish always show anorexia as well as abnormal body color and swimming behavior, causing mortality rates up to 100% (3). NNV is classified in the genus *Betanodavirus* within the family *Nodaviridae*, containing a bipartite positive-sense single-stranded RNA genome. Gene segment RNA1 is about 3,100 nucleotides (nt) in length and encodes the RNA-dependent RNA polymerase (RdRp), while segment RNA2 is approximately 1,400 nt in length and encodes the coat protein (CP). The RNA3 open reading frame (ORF) is also present in RNA1, encoding two nonstructural viral proteins, B1 and B2 (4). According to the variable region of the RNA2 sequence, NNV is divided into four genotypes, tiger puffer nervous necrosis virus (TPNNV), striped jack nervous necrosis virus (SJNNV), barfin flounder nervous necrosis virus (BFNNV), and red-spotted grouper nervous necrosis virus (RGNNV) (5, 6).

Sick hybrid grouper (Epinephelus fuscoguttatus♀ × E. lanceolatus♂) juveniles were collected from an aquaculture fish farm in Liaoning Province, China. Total RNA was isolated from various tissues (eye, brain, and spleen) using TRIzol reagent and reverse transcribed into cDNA using PrimeScript reverse transcriptase according to the manufacturer’s instructions. NNV was diagnosed using PCR with a pair of primers (Table 1) targeting a 632-bp region in the RdRp. RNA1 and RNA2 were amplified using three pairs of overlapping primers and a single pair of primers (Table 1), respectively. Then, the PCR products were subcloned into the pMD-19-T vector (TaKaRa, Japan). To ensure quality, eight positive clones for each overlapping fragment were selected for Sanger sequencing and comparative analysis. RNA1 was assembled with three overlapping fragments.

**TABLE 1:** Primers used for cloning and detection

Name	Sequence (5′–3′)	Expected PCR product (bp)	Comment
RdRp-F	AGCGGGAAATGGAACTGG	632	Detection of NNV
RdRp-R	CGACACGATGTTACGATGC	632	Detection of NNV
JRNV1-F1	TCACTTACGCAAGGTTACCG	1,122	Fragment 1 of RdRp
JRNV1-R1	GACCGGCGAACAGTATCTGAC	1,122	Fragment 1 of RdRp
JRNV1-F2	AGTCTGGGYYTGGARGGC	1,032	Fragment 2 of RdRp
JRNV1-R2	GACGAAAGCRTTDGCAATGC	1,032	Fragment 2 of RdRp
JRNV1-F3	TCCAAGCACCWGCTGT	1,099	Fragment 3 of RdRp
JRNV1-R3	GGGGTGGGAGCRGGCA	1,099	Fragment 3 of RdRp
JRNV2-F1	TCAMAATGGTACGCAARGG	1,363	Fragment of CP
JRNV2-R1	TCACTGCGCGGAGCTAACGGTAAC	1,363	Fragment of CP

After assembly with SeqMan in the DNASTAR software package (Madison, WI, USA), the near-complete RNA1 and RNA2 gene segments were 2,988 nt and 1,319 nt long, respectively. The molecular mass of the RNA-encoding proteins was predicted using EditSeq in the DNASTAR software package. RNA1 encodes the complete RdRp (982 amino acids [aa]) with a calculated molecular mass of 110,474.79 Da, while RNA2 encodes the CP (334 aa) with a calculated molecular mass of 36,472.20 Da.

**FIG 1:**
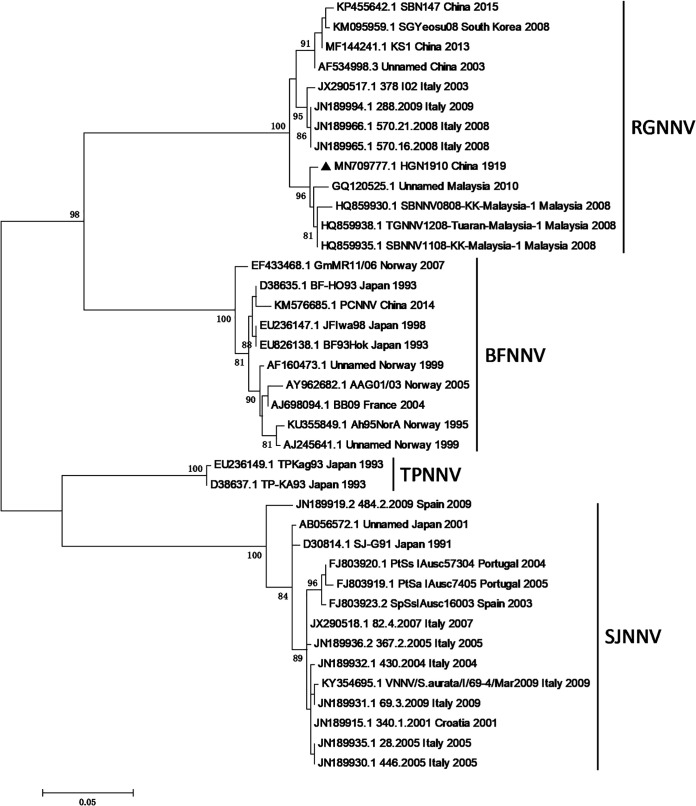
Phylogenetic tree based on the nucleotide sequences of CP genes from 39 NNV isolates. Isolate HGN1910 (GenBank accession number MN709777) is marked with a solid black triangle.

Comparative analyses of the nucleotide sequences were performed in NCBI BLAST using the default parameters and showed that the RNA1 and RNA2 of the NNV strain HGN1910 had the highest identities, 98.41% and 99.49%, with *Betanodavirus* sp. strain LXMC375533 (GenBank accession number MG600032) and TGNNV1208-Tuaran-Malaysia-1 (GenBank accession number HQ859938), respectively. A phylogenetic tree based on the CP gene nucleotide sequences was generated using MEGA 6.0 with the maximum-likelihood method and showed that strain HGN1910 is clustered in the RGNNV genotype (Fig. 1). This genome information will promote further study of the virus diagnostics and the interaction between the host and NNV.

### Data availability.

The complete genome sequence of NNV strain HGN1910 has been deposited in GenBank under the accession numbers MN709776 and MN709777.
